# Supplementary material for: Nasal Staphylococcus aureus carriage promotes depressive behaviour in mice via sex hormone degradation
Source: Nat Microbiol. 2025 Sep 22;10(10):2425–40. doi: 10.1038/s41564-025-02120-6 (PMC12488488; doi:10.1038/s41564-025-02120-6)
Supplement: Supplementary file 1 — Supplementary Tables 1–5 and Supplementary Fig. 1. [file 41564_2025_2120_MOESM1_ESM.pdf]

nature microbiology

Supplementary information

https://doi.org/10.1038/s41564-025-02120-6

Nasal *Staphylococcus aureus* carriage promotes depressive behaviour in mice via sex hormone degradation

In the format provided by the authors and unedited

Supplementary Table 1. Population characteristics

	Healthy	Depressed	P value
**n**	118	100	
**Demographics**			
Sex (Male)	58 (49.2%)	41 (41%)	0.28[1]
Sex (Female)	60 (50.8%)	59 (59%)	
Age	31.1 ± 6.60[2]	29.8 ± 6.78	0.12[3]
**Lifestyles**			
BMI (kg/m^2)	21.6 ± 2.83	21.8 ± 2.95	0.54[3]
**Smoking status**			
Never or not smoked within 3 months	118 (100%)	100 (100%)	
Once or twice a month	0	0	
More than three times a week	0	0	
**Alcohol consumption**			
Never or not drunk within 3 months	118 (100%)	100 (100%)	
Once or twice a month	0	0	
More than three times a week	0	0	
**Clinical information**			
**Severity**			
PHQ-9	1.91 ± 1.41	17.89 ± 5.04	<0.0001[3]
GAD-7	2.02 ± 1.35	14.09 ± 4.94	<0.0001[3]
**Medical history**			
Nasal diseases	0	0	
Oral diseases	0	0	
Family history of any psychiatric disorders	0	0	
Diabetes	0	0	
Hypertension	0	0	
Autoimmune diseases	0	0	
Tumors	0	0	
Pregnancy	0	0	
Lactation	0	0	
**Medication history**			
Antibiotics (within 1 month)	0	0	
Antidepressants (within 3 month)	0	0	
**Serum hormones and cytokines**			
n (available for measurement)	43	76	
FT3 (pmol/l)	4.99 ± 0.62	4.81 ± 0.72	0.11[3]
FT4 (pmol/l)	17.14 ± 1.75	16.59 ± 2.06	0.14[4]
TSH (mIU/l)	1.98 ± 0.97	1.75 ± 0.82	0.18[3]
IFN-α (pg/ml)	2.26 ± 0.61	2.08 ± 0.67	0.15[4]
IFN-γ (pg/ml)	1.84 ± 0.39	1.95 ± 0.52	0.28[3]
IL-10 (pg/ml)	2.41 ± 0.73	2.18 ± 0.59	0.24[3]
IL-17A (pg/ml)	3.5 ± 1.59	3.83 ± 1.76	0.4[3]
IL-1β (pg/ml)	1.07 ± 0.46	1.15 ± 0.64	0.64[3]
IL-4 (pg/ml)	1.84 ± 0.9	1.79 ± 0.71	0.88[3]
IL-5 (pg/ml)	1.8 ± 0.49	1.88 ± 0.6	0.48[3]
IL-6 (pg/ml)	2.67 ± 1.3	2.83 ± 0.87	0.15[3]
TNF-α (pg/ml)	2.23 ± 0.5	2.11 ± 0.74	0.13[3]

[1]Chi-square test
[2]Mean ± SD
[3]Mann-Whitney test
[4]Student's t-test

Supplementary Table 2. Multivariable logistic regression analysis of nasal microbiota diversity and psychosocial-biochemical covariates

	Unadjusted			Model 1			Model 2			Model 3		
	OR	95% CI	P value	OR	95% CI	P value	OR	95% CI	P value	OR	95% CI	P value
Shannon index	0.383	0.20-0.71	**0.003**	0.40	0.20-0.75	**0.005**	0.36	0.16-0.79	**0.012**	0.17	0.033-0.67	**0.017**
Sex (female=1)				1.17	0.65-2.09	0.60	0.90	0.45-1.81	0.78	0.69	0.17-2.64	0.58
Age				0.97	0.93-1.01	0.18	0.99	0.94-1.05	0.84	0.95	0.86-1.06	0.35
BMI (kg/m2)				1.03	0.93-1.14	0.56	0.98	0.86-1.10	0.70	1.01	0.81-1.27	0.90
Financial health (strained=0,balanced=1, comfortable=2)							1.04	0.55-1.97	0.90	0.71	0.21-2.35	0.58
Years of schooling							1.09	0.81-1.49	0.55	1.16	0.77-1.84	0.46
Current partnership status (yes=1)							0.77	0.36-1.65	0.50	0.33	0.061-1.54	0.17
Family relationship self evaluation							0.43	0.28-0.63	**<0.0001**	0.28	0.12-0.59	**0.002**
Adverse childhood experience (yes=1)							3.26	1.15-9.98	**0.030**	0.91	0.12-7.97	0.93
Available supportive friends (3 or more=1)							0.22	0.10-0.44	**<0.0001**	0.19	0.059-0.54	**0.003**
Sleep disorders (yes=1)							2.52	0.97-7.06	0.066	2.64	0.46-19.6	0.30
Serum FT3 (pmol/L)										0.98	0.37-2.64	0.96
Serum FT4 (pmol/L)										0.86	0.61-1.20	0.39
Serum TSH (mIU/L)										0.72	0.36-1.45	0.36
Serum IL-1β (pg/ml)										1.79	0.61-5.83	0.31
Serum IL-6 (pg/ml)										0.80	0.44-1.37	0.421
Serum TNF-α (pg/ml)										0.68	0.26-1.69	0.411
Serum cortisol (ng/ml)										1.00	0.99-1.02	0.67

Supplementary Table 3. Population characteristics for metabolomic sequencing

	Healthy	Depressed	P value
**n**	40	40	
**Demographics**			
Sex (Male)	20 (50%)	20 (50%)	>0.9999[1]
Sex (Female)	20 (50%)	20 (50%)	
Age	32.1 ± 5.54[2]	30.2 ± 7.17	0.18[3]
**Lifestyles**			
BMI (kg/m^2)	21.76 ± 2.63	21.81 ± 2.53	0.92[3]
**Smoking status**			
Never or not smoked within 3 months	40 (100%)	40 (100%)	
Once or twice a month	0	0	
More than three times a week	0	0	
**Alcohol consumption**			
Never or not drunk within 3 months	40 (100%)	40 (100%)	
Once or twice a month	0	0	
More than three times a week	0	0	
**Clinical information**			
**Severity**			
PHQ-9	1.98 ± 1.39	17.27 ± 5.4	<0.0001[4]
GAD-7	1.85 ± 1.53	13.32 ± 5.17	<0.0001[4]
**Medical history**			
Nasal diseases	0	0	
Oral diseases	0	0	
Family history of any psychiatric disorders	0	0	
Diabetes	0	0	
Hypertension	0	0	
Autoimmune diseases	0	0	
Tumors	0	0	
Pregnancy	0	0	
Lactation	0	0	
**Medication history**			
Antibiotics (within 1 month)	0	0	
Antidepressants (within 3 month)	0	0	
**Serum hormones and cytokines**			
n (available for measurement)	27	38	
FT3 (pmol/l)	5.03 ± 0.47	4.86 ± 0.73	0.2[4]
FT4 (pmol/l)	17.47 ± 1.76	16.45 ± 2.29	0.06[3]
TSH (mIU/l)	1.9 ± 1.1	1.76 ± 0.67	0.51[3]
IFN-α (pg/ml)	2.36 ± 0.56	2.02 ± 0.59	0.02[3]
IFN-γ (pg/ml)	1.86 ± 0.26	1.84 ± 0.5	0.84[3]
IL-10 (pg/ml)	2.56 ± 0.6	2.13 ± 0.65	0.05[4]
IL-17A (pg/ml)	3.53 ± 1.26	3.94 ± 1.84	0.5[4]
IL-1β (pg/ml)	1.02 ± 0.32	1.11 ± 0.64	0.97[4]
IL-4 (pg/ml)	1.83 ± 0.99	1.91 ± 0.7	0.4[4]
IL-5 (pg/ml)	1.76 ± 0.45	1.8 ± 0.57	0.74[4]
IL-6 (pg/ml)	2.56 ± 1.5	2.77 ± 0.76	0.07[4]
TNF-α (pg/ml)	2.24 ± 0.44	2.26 ± 0.87	0.63[4]

[1]Chi-square test

[2]Mean ± SD

[3]Student's t-test

[4]Mann-Whitney test

Supplementary Table 4. Putative sex hormone degrading genes

Name	E-value of HMMER	PFAM name	PFAM number	Protein function	Accession number
HSD1	3.6E-26	3-beta hydroxysteroid dehydrogenase/isomerase family	PF01073	Uncharacterized	PQ067567
HSD2	4.50E-20	NmrA-like family	PF05368	Uncharacterized	PQ067568
HSD3	7.80E-06	NAD dependent epimerase/dehydratase family	PF01370	Uncharacterized	PQ106784
HSD4	8.20E-27	short chain dehydrogenase	PF00106	Uncharacterized	PQ067569
HSD5	9.50E-26	NAD dependent epimerase/dehydratase family	PF01370	Uncharacterized	PQ067570
HSD6	7.40E-20	NAD dependent epimerase/dehydratase family	PF01370	Uncharacterized	PQ067571
HSD7	1.40E-16	NAD dependent epimerase/dehydratase family	PF01370	Uncharacterized	PQ106785
HSD8	7.50E-37	short chain dehydrogenase	PF00106	Uncharacterized	PQ067572
HSD9	2.00E-42	short chain dehydrogenase	PF00106	Uncharacterized	PQ067573
HSD10	1.90E-20	NAD dependent epimerase/dehydratase family	PF01370	Uncharacterized	PQ067574
HSD11	5.20E-54	short chain dehydrogenase	PF00106	Uncharacterized	PQ067575
HSD12	2.60E-58	short chain dehydrogenase	PF00106	Uncharacterized	PQ067576
HSD13	1.90E-40	short chain dehydrogenase	PF00106	Uncharacterized	PQ067577
HSD14	1.20E-62	NAD dependent epimerase/dehydratase family	PF01370	Putative UDP-glucose 4-epimerase	PQ067578
HSD15	2.00E-12	NmrA-like family	PF05368	Uncharacterized	PQ067579
HSD16	1.60E-39	NmrA-like family	PF05368	Uncharacterized	PQ106786
butA	3.00E-56	short chain dehydrogenase	PF00106	Diacetyl reductase [(S)-acetoin forming] ButA	PQ067580
capD	1.40E-25	NAD dependent epimerase/dehydratase family	PF01370	Capsular polysaccharide biosynthesis protein CapD	PQ067581
capE	4.30E-27	NAD dependent epimerase/dehydratase family	PF01370	Capsular polysaccharide biosynthesis protein CapE	PQ067582
capF	2.70E-16	NAD dependent epimerase/dehydratase family	PF01370	Capsular polysaccharide biosynthesis protein CapF	PQ067583
capN	8.3E-25	NAD dependent epimerase/dehydratase family	PF01370	Capsular polysaccharide biosynthesis protein CapN	PQ067584
fabG	4.40E-66	short chain dehydrogenase	PF00106	3-oxoacyl-[acyl-carrier-protein] reductase FabG	PQ067585

fabI	1.60E-19	short chain dehydrogenase	PF00106	Enoyl-[acyl-carrier-protein] reductase [NADPH] FabI	PQ067586

Supplementary Table 5. Oligonucleotides

Primer name	Sequence (5'-3')	Usage
HSD1-F-BamH1	GAGGGATCCATGAATAATAAAGTATTAGTG	Heterologous expression of *hsd*1 using pET28a vector
HSD1-R-EcoR1	GACGAATTCCGTATTAAATGAAATTTAGCC	
HSD2-F-EcoR1	GAGGAATTCATGAATATTATATTAACAGGTG	Heterologous expression of *hsd*2 using pET28a vector
HSD2-R-HindIII	GAGAAGCTTAATATTTTCTTGTAAAAATG	
HSD3-F-BamH1	GAGGGATCCATGAAACCTAAAGTTTTA	Heterologous expression of *hsd*3 using pET28a vector
HSD3-R-HindIII	GAGAAGCTTTTTAGTATATTTCATATTTTCT	
HSD4-F-BamH1	GAGGGATCCATGAAAGGACAACATTTTA	Heterologous expression of *hsd*4 using pET28a vector
HSD4-R-HindIII	GAGAAGCTTTGCTTTATTTTTAAATAAT	
HSD5-F-BamH1	GAGGGATCCATGAAAAAAATTATGATTAC	Heterologous expression of *hsd*5 using pET28a vector
HSD5-R-EcoR1	GACGAATTCGTTATTATTTTTAACAGTATC	
HSD6-F-BamH1	GAGGGATCCATGAAACAATACTTAATTAC	Heterologous expression of *hsd*6 using pET28a vector
HSD6-R-SalI	GAGGTCGACCTTACTAATTAAATCTTCAAG	
HSD7-F-BamH1	GAGGGATCCATGAAAGAAGGACTTTTT	Heterologous expression of *hsd*7 using pET28a vector
HSD7-R-EcoR1	GACGAATTCCTTATTGAATATTGTTTT	
HSD8-F-BamH1	GAGGGATCCATGAAAGCATTAGTATTAGG	Heterologous expression of *hsd*8 using pET28a vector
HSD8-R-HindIII	GAGAAGCTTAATATACCAAGCACCATTAA	
HSD9-F-BamH1	GAGGGATCCATGGCAGCTCAAGATCCTAG	Heterologous expression of *hsd*9 using pET28a vector
HSD9-R-EcoR1	GACGAATTCATCTATTTGCACTCCACCGC	
HSD10-F-BamH1	GAGGGATCCATGAGTAAAATTTTTGTAAC	Heterologous expression of *hsd*10 using pET28a vector
HSD10-R-HindIII	GAGAAGCTTTTTAATTGGATTCATGCCAT	
HSD11-F-BamH1	GAGGGATCCATGAAACGTTTGGAAAAT	Heterologous expression of *hsd*11 using pET28a vector
HSD11-R-SalI	GAGGTCGACTTCCAATGTCCGCTTCCA	
HSD12-F-BamH1	GAGGGATCCATGACAGTATTAACAGAT	Heterologous expression of *hsd*12 using pET28a vector
HSD12-R-HindIII	GAGAAGCTTTACAGGACGCACTGTAAT	
HSD13-F-BamH1	GAGGGATCCATGACTAAAATTGTGTTA	Heterologous expression of *hsd*13 using pET28a vector
HSD13-R-HindIII	GAGAAGCTTCCAAGGCATTTCACCATT	
HSD14-F-BamH1	GAGGGATCCTTGGAAAAAGTTTTGATAAC	Heterologous expression of *hsd*14 using pET28a vector
HSD14-R-HindIII	GAGAAGCTTCGACATTTCCACTTCTTTCG	
HSD15-F-BamH1	GAGGGATCCATGAATATTTTGGTTATAGG	Heterologous expression of *hsd*15 using pET28a vector

HSD15-R-HindIII	GAGAAGCTTATCCGTTTCATTTTCAAATT	
HSD16-F-BamH1	GAGGGATCCATGAAAGATATTTTAGTAAT	Heterologous expression of *hsd*16 using pET28a vector
HSD16-R-SalI	GAGGTCGACCTTATTATAGTCTTGGACT	
HSD12-up-F	TACTTCCAATCCAATGTCACCTCTATTATTAGTGTA	Construction of *S. aureus* *hsd*12 deletion mutant
HSD12-up-R	AAATGTGTTTGAGGTGTGGG	
HSD12-down-F	CCCACACCTCAAACACATTTAATGATCGACTCCTTTGAT	
HSD12-down-R	TTATCCACTTCCAATGGTAAGTAGCTATGATATTTAC	
HSD12-ko.test-F	CGGCATAAATAAAGTTCCCTTCA	Verification of *S. aureus hsd*12 deletion mutant
HSD12-ko.test-R	GCGTTCATTCATGCCATTATGC	
HSD12-F	GGTGCAGGTAGTGGTATTGG	Amplification of the *hsd*12 gene in *S. aureus* strains
HSD12-R	AGGACGCACTGTAATTTCATTCA	
ST88-A	TACTTCCAATCCAATGGTAAGTAGCTATGATATTTAC	Construction of *hsd*12 deletion mutant
ST88-B	AATGATCGACTCCTTTGAT	
ST88-C	ATCAAAGGAGTCGATCATTAAATGTGTTTGAGGTATGGG	
S88-D	TTATCCACTTCCAATGTTTTCGACATCTATCACCAG	
nuc-RT-F	AGCGATTGATGGTGATAC	Absolute quantification of *S aureus*
nuc-RT-R	CGCTAAGCCACGTCCATATT	
Tph2-RT-F	GGTTGTCCTTGGATTCTGCTG	qPCR test for TPH2
Tph2-RT-R	GCCTGGATTCGATATGAAGCAT	
Th-RT-F	CACGTCCCCAAGGTTCATTG	qPCR test for TH
Th-RT-R	CGAGACAGTGAGGAGGGTTT	
Ddc-RT-F	TAGCTGACTATCTGGATGGCAT	qPCR test for DDC
Ddc-RT-R	GTCCTCGTATGTTTCTGGCTC	
Gapdh-RT-F	AGGTCGGTGTGAACGGATTTG	qPCR test for GAPDH
Gapdh-RT-R	TGTAGACCATGTAGTTGAGGTCA	

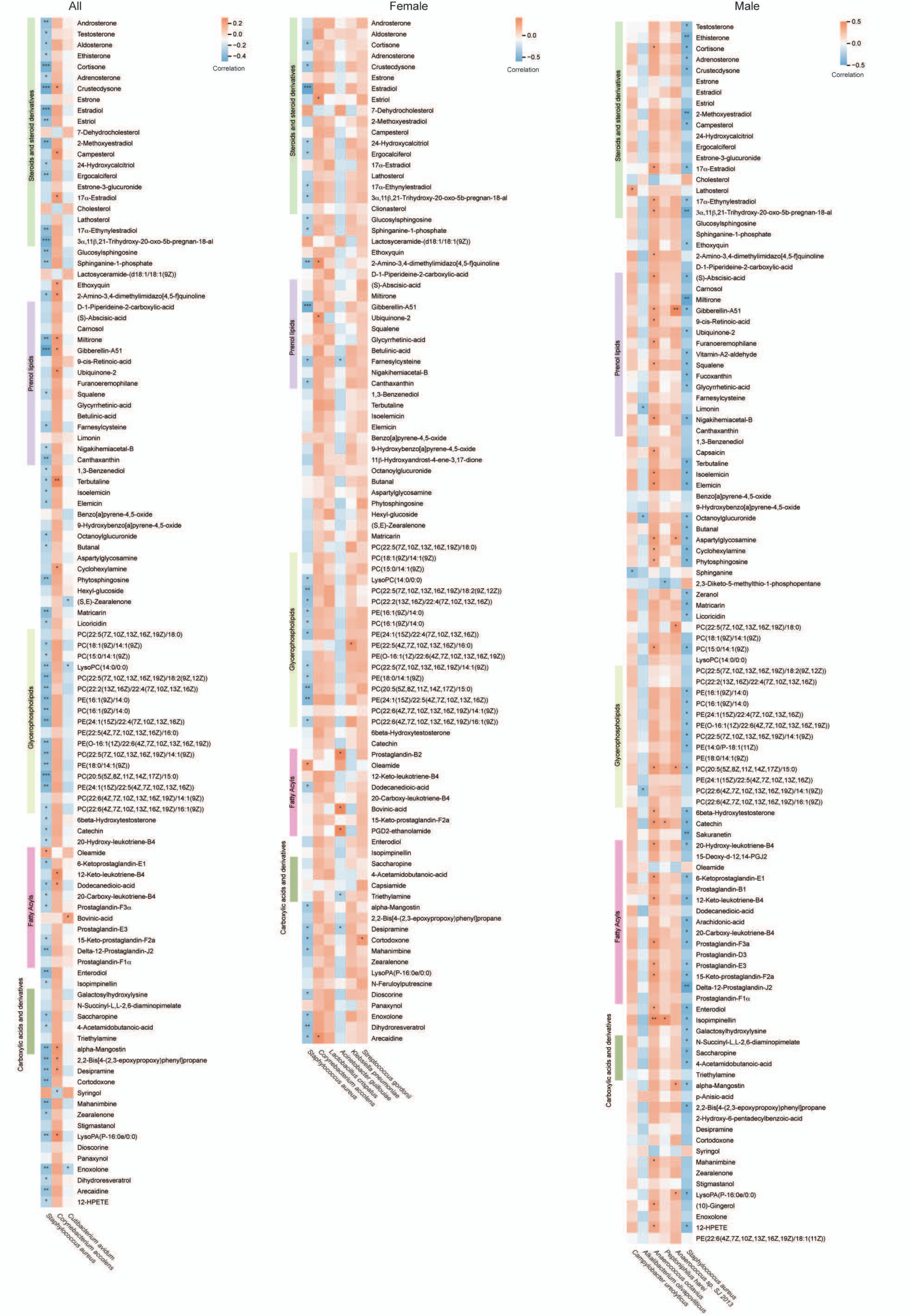

Supplementary Figure 1. HAllA-based correlation analysis of species-metabolite interactions. Hierarchical all-against all (HAllA) correlation analysis was performed between the counts of species and metabolites to evaluate associations between significantly differed species (FDR < 0.05 in Maaslin2 results of group variables) and metabolites [FDR<0.05 and VIP (Variable Importance for the Projection)>1]. *, P<0.05; **, P<0.01; ***, P<0.001.
