# Supplementary material for: Nasal Staphylococcus aureus carriage promotes depressive behaviour in mice via sex hormone degradation
Source: Nat Microbiol. 2025 Sep 22;10(10):2425–40. doi: 10.1038/s41564-025-02120-6 (PMC12488488; doi:10.1038/s41564-025-02120-6)
Supplement: Supplementary file 2 — Reporting Summary [file 41564_2025_2120_MOESM2_ESM.pdf]

nature portfolio

Corresponding author(s): Michael Otto

Last updated by author(s): Jun 9, 2025

Reporting Summary

Nature Portfolio wishes to improve the reproducibility of the work that we publish. This form provides structure for consistency and transparency in reporting. For further information on Nature Portfolio policies, see our Editorial Policies and the Editorial Policy Checklist.

Statistics

For all statistical analyses, confirm that the following items are present in the figure legend, table legend, main text, or Methods section.

n/a	Confirmed	
☐	☒	The exact sample size (n) for each experimental group/condition, given as a discrete number and unit of measurement
☐	☒	A statement on whether measurements were taken from distinct samples or whether the same sample was measured repeatedly
☐	☒	The statistical test(s) used AND whether they are one- or two-sided<br>*Only common tests should be described solely by name; describe more complex techniques in the Methods section.*
☐	☒	A description of all covariates tested
☐	☒	A description of any assumptions or corrections, such as tests of normality and adjustment for multiple comparisons
☐	☒	A full description of the statistical parameters including central tendency (e.g. means) or other basic estimates (e.g. regression coefficient) AND variation (e.g. standard deviation) or associated estimates of uncertainty (e.g. confidence intervals)
☐	☒	For null hypothesis testing, the test statistic (e.g. F, t, r) with confidence intervals, effect sizes, degrees of freedom and P value noted<br>*Give P values as exact values whenever suitable.*
☒	☐	For Bayesian analysis, information on the choice of priors and Markov chain Monte Carlo settings
☒	☐	For hierarchical and complex designs, identification of the appropriate level for tests and full reporting of outcomes
☐	☒	Estimates of effect sizes (e.g. Cohen's d, Pearson's r), indicating how they were calculated

Our web collection on statistics for biologists contains articles on many of the points above.

Software and code

Policy information about availability of computer code

Data collection	Cultured bacterial species were identified by MALDI-TOF-MS (Bruker Daltonics, Bremen, Germany) using MALDI Biotyper Compass software v4.1 (Bruker Daltonics).<br>Full-length 16S rRNA gene sequencing was performed using the PacBio platform with SMRT Link software v11.0 (Pacific Biosciences).<br>Whole genome sequencing and RNA-seq were performed using the Illumina NovaSeq 6000 platform with Illumina Control Software v1.7 and Real-Time Analysis (RTA) v3.4.4 (Illumina).<br>LC-MS/MS analyses of nasal metabolomics were performed using a UHPLC system (Vanquish, Thermo Fisher Scientific) coupled to an Orbitrap Exploris 120 mass spectrometer with Xcalibur software v4.3 (Thermo Fisher Scientific).<br>LC-MS/MS analysis of steroid hormones was performed using the Waters ACQUITY UPLC / Xevo TQ-S mass spectrometer with MassLynx software v4.2 (Waters).<br>LC-MS/MS analysis of neurotransmitters was performed using the Waters ACQUITY Premier / SCIEX Triple Quad™ 6500+ mass spectrometer with SCIEX OS software v3.0 (SCIEX).<br>Mice behavior was recorded and analyzed using VisuTrack software v3.0 (Shanghai XinRuan Information Technology Co., Ltd.).<br>qRT-PCR data were obtained on an ABI 7500 thermocycler (Applied Biosystems) using 7500 Software v2.3 (Applied Biosystems).<br>Absorbance was detected using the BioTek Synergy2 microplate reader with Gen5 software v3.08 (BioTek Instruments).<br>A NanoZoomer S210 digital slide scanner was used to capture tissue paraffin sections for H&E staining with NDP.scan software v3.2 (Hamamatsu Photonics).
Data analysis	Full-length 16S rRNA gene sequencing data was performed using the DADA2 workflow in the QIIME2 software pipeline. The taxonomy of amplicon sequence variants (ASVs) sequences was analyzed using RDP Classifier version 2.13 against the NT_16S (v20221012) database.<br>Whole-genome sequencing data were assembled using SPAdes v3.15.4, and MLST was identified using mlst v2.23.0 (https://github.com/tseemann/mlst).

The raw non-targeted metabolomics data were converted to the mzXML format using ProteoWizard and processed with an XCMS R package (v3.22.0), for peak detection, extraction, alignment, and integration. An in-house MS2 database (BiotreeDB) was used for metabolite annotation. Waters MassLynx V4.1 was employed for steroid hormones LC-MS/MS data acquisition and processing. SCIEX Analyst Work Station Software (Version 1.6.3) was employed for neurotransmitters LC-MS/MS data processing.
The RNA-seq data were analyzed using the HISAT2 (v2.2.1)-featureCounts (v2.0.3)-edgeR (v3.40.2) pipeline.
The Kyoto Encyclopedia of Genes and Genomes (KEGG) database was used to identify enriched pathways.
SDR superfamily members were identified using hmmer v3.0, by scanning protein sequences of the P24-2 (ST398) S. aureus genome.
Principal coordinate analyses (PCoA) and Orthogonal Partial Least Squares-Discriminant Analysis (OPLS-DA) were performed and evaluated using vegan and ropls R package (v1.30.0). qRT-PCR data and absorbance data were analyzed in Excel (Microsoft Office 2019). All other analysis methods are presented in the manuscript. Unless otherwise specified, statistical analysis was performed using Prism 10.2.0 software.

For manuscripts utilizing custom algorithms or software that are central to the research but not yet described in published literature, software must be made available to editors and reviewers. We strongly encourage code deposition in a community repository (e.g. GitHub). See the Nature Portfolio guidelines for submitting code & software for further information.

Data

Policy information about availability of data

All manuscripts must include a data availability statement. This statement should provide the following information, where applicable:

- Accession codes, unique identifiers, or web links for publicly available datasets
- A description of any restrictions on data availability
- For clinical datasets or third party data, please ensure that the statement adheres to our policy

Raw microbiome sequencing and transcriptome data have been deposited in the NCBI's SRA database under Bioproject number PRJNA1138490. Raw metabolomics data have been deposited in the MetaboLights database under accession number MTBLS10742. The nucleotide sequences of putative HSD proteins identified in this study have been deposited in NCBI's GenBank database under accession number PQ067567 to PQ067586, and PQ106784 to PQ106786. All other data are presented in this manuscript. Source data files (see Supporting Material) contain results for all figures with quantitative data.

Research involving human participants, their data, or biological material

Policy information about studies with human participants or human data. See also policy information about sex, gender (identity/presentation), and sexual orientation and race, ethnicity and racism.

Reporting on sex and gender	Both male (n=58, healthy cohort; n=41, depressed cohort) and female (n=60, healthy cohort; n=59, depressed cohort) participants aged between 18 and 44 year were included. There was no significant difference in sex between health and depression groups.
Reporting on race, ethnicity, or other socially relevant groupings	All participants were Han Chinese.
Population characteristics	In average, the healthy participants were 31.1 ± 6.60 years old, and the depression participants 29.8 ± 6.78 years old. There was no significant difference in age between health and depression groups.
Recruitment	Depressive patients and healthy volunteers were enrolled between November 2022 to November 2024. Healthy volunteers were recruited from the Physical Examination Center of Renji Hospital. Depression patients were recruited from the Department of Psychological Medicine of Renji Hospital. Each participant was provided with a detailed written questionnaire by the research physicians. All participants were between 18 and 44 years of age. Depression patients were diagnosed according to DSM-IV by clinical psychologists and had no other psychiatric disorders or family history of any psychiatric disorders. The severity of depression and anxiety was evaluated using Patient Health Questionnaire-9 (PHQ-9) and Generalized Anxiety Disorder-7 (GAD-7) scores, respectively. Healthy controls were physically healthy and lacked any neurological illness or related family history, with PHQ-9 and GAD-7 scores both below 4. Other exclusion criteria for both control and depression groups included nasal or oral diseases, previous use of any type of antidepressant medication within the past three months, use of antimicrobial drugs within the past four weeks, thyroid dysfunction, diabetes, hypertension, autoimmune diseases, tumors, pregnancy, or lactation.
Ethics oversight	The human clinical study was approved by the ethics committee of Renji Hospital, Shanghai Jiao Tong University School of Medicine, Shanghai, China (approval number KY2022-139-B). Informed consent was obtained from all human research participants.

Note that full information on the approval of the study protocol must also be provided in the manuscript.

Field-specific reporting

Please select the one below that is the best fit for your research. If you are not sure, read the appropriate sections before making your selection.

☒ Life sciences ☐ Behavioural & social sciences ☐ Ecological, evolutionary & environmental sciences

For a reference copy of the document with all sections, see nature.com/documents/nr-reporting-summary-flat.pdf

Life sciences study design

All studies must disclose on these points even when the disclosure is negative.

Sample size	No statistical methods were used to pre-determine sample sizes but our sample sizes were chosen based on those reported in previous publications. For the human microbiome study, the number of analyzed individuals were consistent with previous study analyzing nasal microbiomes (reference: Nat Microbiol. 2023 Feb;8(2):218-230.).<br>For non-targeted metabolomics analysis, sample sizes were consistent to previous study analyzing nasal metabolites (reference: Sci Rep. 2022 Jun 15;12(1):10029.).<br>For animal studies, our sample sizes were chosen based on those reported data in previous publications (references: Cell Metab. 2023 Apr 4;35(4):685-694.e5. and Cell Host Microbe. 2024 Feb 14;32(2):227-243.e6.).<br>For in vitro experiments, at least three replicates were performed, and deemed sufficient to achieve reliable results.
Data exclusions	In amplicon sequence analysis, to mitigate potential contamination effects, all ASVs detected in the negative controls were removed from subsequent analyses. Sequences with a quality score of less than three or expected errors greater than two were filtered out, and only ASV sequences present in at least two samples were retained to eliminate spurious features.<br>In non-targeted metabolomics analysis, the cutoff for peak annotation was set at 0.3 and only metabolites identified in MS2 level were used for analysis.<br>In heterologous expression of putative SDR protein in E. coli, putative SDR proteins were identified using HMMER v3.0 with an E-value below 1e-5. The sequences of putative SDR superfamily proteins were compared using blastp with the Uniprot database to exclude candidates with known other functions.
Replication	All in vitro sex hormones degrading experiments were performed with at least three replicates deemed sufficient to achieve reliable results, and these experiments have been validated multiple times and similar results have been obtained. Other experiments were not repeated, as they were all performed with a number of replicates deemed sufficient to achieve reliable results.
Randomization	In culture-based analysis, twenty-four random colonies were isolated and identified by MALDI-TOF-MS in each sample.<br>For nasal microbiota transplantation experiments, donor nasal microbiota samples were randomly selected from health and depression groups.<br>For non-targeted metabolomics analysis, metabolites were determined in the noses of n=40 randomly selected individuals from health and depression groups, respectively.<br>Sequence type distribution was analyzed using randomly selected S. aureus isolates (one per colonized individual; n=31, healthy cohort; n=45, depressed cohort).<br>Conversion of estradiol to estrone and testosterone to androstenedione were determined by culture filtrates from randomly selected different nasal bacteria (n=3/group) and randomly selected S. aureus isolates of main isolate STs (n=3/group).<br>Mice used in the experiments were litter mates, sex and age-matched, and randomized into control and experimental groups.
Blinding	For animal experiments, mouse tissue paraffin sections were used for H&E, IF and IHC. Slides were examined independently by a histopathologist who was blinded to the treatment. Blinding was not performed in other experiments.

Reporting for specific materials, systems and methods

We require information from authors about some types of materials, experimental systems and methods used in many studies. Here, indicate whether each material, system or method listed is relevant to your study. If you are not sure if a list item applies to your research, read the appropriate section before selecting a response.

Materials & experimental systems

n/a	Involved in the study	
☐	☒	Antibodies
☒	☐	Eukaryotic cell lines
☒	☐	Palaeontology and archaeology
☐	☒	Animals and other organisms
☒	☐	Clinical data
☒	☐	Dual use research of concern
☒	☐	Plants

Methods

n/a	Involved in the study	
☒	☐	ChIP-seq
☒	☐	Flow cytometry
☒	☐	MRI-based neuroimaging

Antibodies

Antibodies used	Primary antibodies:<br>Anti-IBA1 antibody (Guinea pig polyclonal antibody, Oasis biofarm, Catalog: OB-PGP049) was used to determine IBA1 at a dilution of 1:100.<br>Anti-Olfactory Marker Protein antibody (Rabbit polyclonal antibody, Bioss, Catalog: bs-19568R) was used to determine Olfactory Marker Protein at a dilution of 1:100.<br>Anti-Ly6G antibody (Rabbit monoclonal antibody, Abcam, Catalog: ab238132, Clone: EPR22909-135) was used to stain Neutrophils at a dilution of 1:500.<br>Secondary antibodies:<br>Goat anti-rabbit IgG, HRP-linked Antibody, Abcam, Catalog: ab205718, Antibody Dilution: 1:2000.

Alexa Fluor 594 goat-anti-guinea pig IgG, Oasis biofarm, Catalog: G-GP594, Antibody Dilution: 1:200.
Alexa Fluor 488 donkey anti-Rabbit IgG, ThermoFisher, Catalog: A21206, Antibody Dilution: 1:400.

Validation

Primary antibodies:
Anti-IBA1 antibody (Guinea pig polyclonal antibody, Catalog: OB-PGP049, RRID: AB_2934253)
https://www.oasisbiofarm.net/#/productdetails?proId=bd7ab575ebfa41009108908905941dd0&exp2=%E6%8A%97%E4%BD%93
Anti-Olfactory Marker Protein antibody
http://www.bioss.com.cn/prolook_03.asp?id=AF08169606023114&pro37=1
Anti-Ly6G antibody (Rabbit monoclonal antibody, Abcam, Catalog: ab238132, Clone: EPR22909-135)
https://www.abcam.cn/products/primary-antibodies/ly6g-antibody-epr22909-135-ab238132.html
Secondary antibodies:
Goat anti-rabbit IgG, HRP-linked Antibody, Abcam, Catalog: ab205718, Antibody Dilution: 1:2000.
https://www.abcam.cn/products/secondary-antibodies/goat-rabbit-igg-hl-hrp-ab205718.html
Alexa Fluor 594 goat-anti-guinea pig IgG, Oasis biofarm, Catalog: G-GP594.
https://www.oasisbiofarm.net/#/productdetails?proId=f823c846c54442509e2f9ea914a55b29&exp2=%E4%BA%8C%E6%8A%97
Alexa Fluor 488 donkey anti-Rabbit IgG, ThermoFisher, Catalog: A21206.
https://www.thermofisher.cn/cn/zh/antibody/product/Donkey-anti-Rabbit-IgG-H-L-Highly-Cross-Adsorbed-Secondary-Antibody-Polyclonal/A-21206

Animals and other research organisms

Policy information about studies involving animals; ARRIVE guidelines recommended for reporting animal research, and Sex and Gender in Research

Laboratory animals

C57BL/6J specific pathogen-free (SPF) mice were purchased from GemPharmatech and bred in-house under SPF conditions. Mice were provided with food and water ad libitum and housed at consistent ambient temperature (22 ± 1°C) and humidity (50%±5%) with a 12-h light–dark cycle.

Wild animals

Study did not involve wild animals.

Reporting on sex

Both male and female C57BL/6J mice aged 6 weeks were used for experiments. Sex was considered in the study design because we found that sex hormones were reduced in the nasal cavity of depression people of both sexes and were related to increased abundance of S. aureus. Both male and female C57BL/6J mice received consistent nasal microbiota transplantation or nasal bacteria colonization operations. Both male and female C57BL/6J mice underwent consistent behavior tests and were used for RNA-seq, precursors of neurosteroids and neurotransmitter detection. Some male mice were additionally exposed to chronic unpredictable mild stress (CUMS). Testosterone levels were determined in the nose and brain tissues of male mice, while estradiol levels were determined in the nose and brain tissues of female mice.

Field-collected samples

Study did not involve field-collected samples.

Ethics oversight

All animal procedures followed the ethical guidelines outlined in the Guide for the Care and Use of Laboratory Animals proposed by the Institute for Laboratory Animal Research of the National Academy of Sciences, and all protocols were approved by the Animal Welfare Committee of Renji Hospital, Shanghai Jiao Tong University School of Medicine, Shanghai, China (approval number RJ2023-025B).

Note that full information on the approval of the study protocol must also be provided in the manuscript.

Plants

Seed stocks

Not Applicable

Novel plant genotypes

Not Applicable

Authentication

Not Applicable
